# Immuno-Metabolism and Microenvironment in Cancer: Key Players for Immunotherapy

**DOI:** 10.3390/ijms21124414

**Published:** 2020-06-21

**Authors:** Gaia Giannone, Eleonora Ghisoni, Sofia Genta, Giulia Scotto, Valentina Tuninetti, Margherita Turinetto, Giorgio Valabrega

**Affiliations:** Candiolo Cancer Institute, FPO—IRCCS, University of Turin, 10060 Torino, Italy; gaia.giannone@ircc.it (G.G.); gentasofia@gmail.com (S.G.); giulia.scotto@ircc.it (G.S.); valentina.tuninetti@ircc.it (V.T.); margherita.turinetto@ircc.it (M.T.); giorgio.valabrega@ircc.it (G.V.)

**Keywords:** immunotherapy, metabolism, ICI resistance, macrophages, antiangiogenic, adenosine pathway, IDO inhibitors, PI3K/ALK pathway, microbiome

## Abstract

Immune checkpoint inhibitors (ICIs) have changed therapeutic algorithms in several malignancies, although intrinsic and secondary resistance is still an issue. In this context, the dysregulation of immuno-metabolism plays a leading role both in the tumor microenvironment (TME) and at the host level. In this review, we summarize the most important immune-metabolic factors and how they could be exploited therapeutically. At the cellular level, an increased concentration of extracellular adenosine as well as the depletion of tryptophan and uncontrolled activation of the PI3K/AKT pathway induces an immune-tolerant TME, reducing the response to ICIs. Moreover, aberrant angiogenesis induces a hypoxic environment by recruiting VEGF, T_reg_ cells and immune-suppressive tumor associated macrophages (TAMs). On the other hand, factors such as gender and body mass index seem to affect the response to ICIs, while the microbiome composition (and its alterations) modulates both the response and the development of immune-related adverse events. Exploiting these complex mechanisms is the next goal in immunotherapy. The most successful strategy to date has been the combination of antiangiogenic drugs and ICIs, which prolonged the survival of patients with non-small-cell lung cancer (NSCLC) and hepatocellular carcinoma (HCC), while results from tryptophan pathway inhibition studies are inconclusive. New exciting strategies include targeting the adenosine pathway, TAMs and the microbiota with fecal microbiome transplantation.

## 1. Introduction

The use of immune checkpoint inhibitors (ICIs)—monoclonal antibodies targeting programmed death protein 1 (PD-1), programmed death protein 1 ligand (PD-L1) and CTLA-4—has yielded impressive results in many settings and is currently a cornerstone in the treatment of metastatic melanoma, non-small-cell lung carcinoma (NSCLC), renal cell carcinoma (RCC), urothelial carcinoma and Hodgkin’s lymphoma (HL) [1,2,3,4,5]. However, the clinical benefit is not uniform in all cancer types, ranging between an overall response rate (ORR) of 60–80% in relapsed/refractory Hodgkin lymphoma (HL) to less than 20% in ovarian cancer, prostate cancer and hormone positive breast cancer (BC), being the prospects for employing ICIs across a broader spectrum of tumors elusive so far [6,7]. Furthermore, clinical follow-up results have shown that a significant percentage of patients who are, at first, responders eventually relapse with drug-resistant disease [8,9,10].

**Figure 1:**
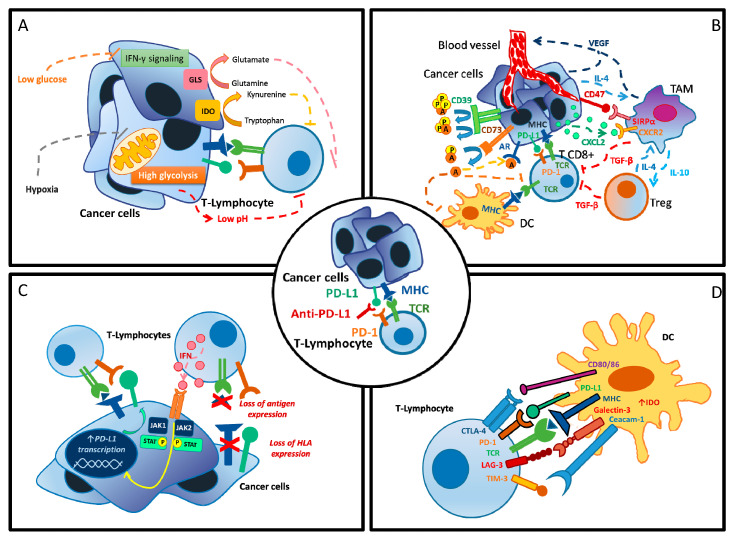
Mechanisms of resistance to PD-1/PD-L1 inhibition. (**A**) Metabolic dysregulation at cellular level. (**B**) Altered tumor microenvironment. (**C**) Tumor cell adaptation. (**D**) Activation of alternative immune checkpoint. A = adenosine, AR = androgen receptor, CTLA-4 = cytotoxic T-lymphocyte antigen 4, CXCL2 = C-X-C Motif Chemokine Ligand 2, CXCR2 = C-X-C Motif Chemokine Receptor 2, CD39 = ecto-nucleoside triphosphate diphosphohydrolase 1, CD47 = integrin-associated protein, CD80/86 = B7 protein, DC = dendritic cell, GLS = glutaminase, HLA = human leucocyte antigen, IL-4 = interleukin 4, IL-10 = interleukin 10, IDO = indoleamine 2,3-dioxygenase, IFN-γ = interferon gamma, JAK1 = Janus kinase 1, JAK2 = Janus kinase 2, LAG-3 = lymphocyte-activation gene 3, MHC = major histocompatibility complex, P = phosphate, PD-1 = protein cell death 1, PD-L1 = protein cell-death ligand 1, STAT-3 = signal transducer and activator of transcription 3, SIRPα = signal regulatory Protein alpha, TAM = tumor associated macrophages, TCR = T-cell receptor, TGF-β = transforming growth factor beta, Treg = regulatory T cells, VEGF = vascular endothelial growth factor.

The mechanisms occurring in primary and secondary resistant tumors are complex and involve both the tumor and the host itself (Figure 1) [10]. Among them, metabolic alterations that modify the tumor microenvironment (TME) and the physiological processes of the host play a central role in the development of resistance to ICIs [10,11,12,13,14].

In this review, we describe key tumor and host metabolic pathways involved in resistance to ICIs. Moreover, we focus on possible strategies to overcome primary and acquired resistance and to improve the response to ICIs.

We used the mesh keywords “immune-resistance”, “metabolism”, “immune checkpoints inhibitors” and “cancer”, manually selecting the most relevant papers in this field. We thus performed research on clinicaltrial.gov, selecting key ongoing clinical trials targeting immune metabolism

## 2. Resistance to ICIs: Immune Metabolism Dysregulation at Cellular Level

Multiple mechanisms such as the overexpression of immune checkpoint molecules, loss of nutrients through vasculature impairment and dysregulation of metabolic pathways were recently shown to affect T cell recruitment and metabolic activities in the TME [15,16,17,18]. These alterations, indeed, can put immune cells into a hypo-responsive state, resulting in T cell exhaustion and anergy, impairing the immune control of tumor growth and the response to ICIs [19,20,21]. Moreover, a great deal of attention has recently been given to the role of macrophages and myeloid-derived suppressor cells (MDSC) as mediators of tumor-associated immune suppression and ICI-resistance [22,23].

### 2.1. Inhibitors of the Adenosine Pathway

Adenosine, generated from adenosine-tri-phosphate (ATP) by the ectonucleotidases CD39^+^ and CD73^+^, is a well-described anti-inflammatory modulator of the immune response in peripheral tissues [24]. In inflamed and damaged tissues (i.e., cancers), extracellular adenosine can increase in concentration by up to 100 times and accumulate, inhibiting the effector functions of various immune cell populations—including CD8^+^ T cells, natural killer (NK) cells, dendritic cells (DC) and macrophages—but also enhance the proliferation and polarization of immunosuppressive cells, thereby promoting the progression of neoplasms [25].

There are four G protein-coupled adenosine receptors (ARs): A1R, A2AR, A2BR and A3R [26]. Once activated, ARs induce the differentiation of monocytes to macrophages. Moreover, they stimulate the immune tolerance by promoting activity of macrophages and affecting their production of IL-12 and TNFα [27,28]. Adenosine also influences neutrophils’ activity: it reduces their adherence, transmigration and degranulation, finally causing an inhibition of Fc receptor-mediated phagocytosis and superoxide production [29,30,31].

Moreover, it has been demonstrated that CD73^+^ tumor cells are resistant to PD-1 blockade and that the simultaneous blockade of CD73^+^ and PD-1^+^ could augment intra-tumoral CD8^+^ infiltration and enhance tumor control and survival in mice [32,33].

Several drugs such as those targeting CD39^+^ and CD73^+^ (that generate adenosine) and anti-AR agents (that consume adenosine) have been proven efficacious in preclinical studies and mouse tumor models [34].

Of note, the concurrent inhibition of CD39 and CD73 failed to potentiate the suppression of adenosine production in cancer cell lines [35].

Novel antibodies and small molecules targeting this pathway are now under investigation in early-phase clinical trials enrolling patients with advanced cancer and may be combined with standard chemotherapy and checkpoint inhibitors to revert immune resistance [36,37] (Table 1).

Recently, results from a Phase 1 trial exploring the combination of oleclumab (anti-CD73) and durvalumab were reported by Overman et al. [38]. Combination therapy showed encouraging clinical activity in pancreatic cancer (pancr) and, potentially, in colorectal cancer (CRC). Partial Response (PR) was observed for 1/21 CRC and 2/20 pancr patients; stable disease (SD) was observed in 2/21 CRC and 3/20 pancr patients, respectively. The most frequent toxicities in the expansion cohort were diarrhea (8.7%), pyrexia (8.7%), fatigue (6.5%), and alanine aminotransferase (ALT) (6.5%), aspartate aminotransferase (AST) (6.5%), and ALP increases (6.5%) [38].

### 2.2. Inhibitors of Angiogenesis Pathways

The TME is a complex system composed of both blood and lymphatic vessels and filled by immune cells (resident DCs, MDSCs, tumor-associated macrophages (TAMs) and resident and infiltrating T cells, including regulatory T cells (T_reg_)) but also stromal cells and CAFs (cancer activated fibroblasts) [39,40]. The cancer vasculature is aberrant both structurally and functionally and thus favors an hypoxic environment with a low pH and high interstitial pressure due to alternative lymphatic drainage, which in turn cause an immune-tolerant setting [39,40]. In this context, T_reg_ cells, TAMs and altered endothelial cells (ECs) replicate, while there is a reduction in DC maturation. All these changes induce altered antigen presentation and inhibit the migration of CD8^+^ T cells into the tumor core [41,42,43]. Furthermore, the abnormal TME lead TAMs to polarize from an anticancer M1-like phenotype towards an immunosuppressive M2 phenotype

Importantly, there is an upregulation of the PD-1/PD-L1 pathway both in immune cells and tumor cells, thus exhausting this mechanism. Lastly, from a biohumoral point of view, the vascular endothelial growth factor (VEGF) both induces the growth of MDSCs and enhances their immune-modulatory activity [44,45].

Thus, drugs that modify the TME might improve the effectiveness of immunotherapy.

Antiangiogenic agents can alleviate immunosuppression, enabling CD8^+^ T cells to access the tumor tissue by relieving endothelial anergy and increasing adhesion molecule expression, thus augmenting checkpoint blockade activity [46,47].

Several clinical trials evaluating the combination of anti-PD-L1, anti-PD-1 or anti-CTLA-4 agents with anti-VEGF therapies (above all, bevacizumab) have been conducted in multiple tumor types and in different settings.

Given the number of these studies, we report key trials with available results (Table 2).

Of note, the anti-PD-L1 agent atezolizumab plus bevacizumab in combination with carboplatin and paclitaxel has already received FDA approval for the treatment of metastatic non-squamous NSCLC with no epidermal growth factor receptor (EGFR) or anaplastic lymphoma kinase (ALK) genomic tumor aberration based on the *IMpower 150* results [48,49]. Regulatory approval is also under evaluation for the treatment of patients with unresectable hepatocellular carcinoma (HCC) not receiving prior systemic therapy, according to the *IMbrave 150* study [50,51].

Additional trials evaluating anti-PD-L1 agents plus bevacizumab alone or as part of a larger regimen are actually recruiting in multiple cancer settings including CRC (NCT03869190 and NCT04068610), ovarian cancer (NCT03353831, NCT03806049, NCT03737643 and NCT03596281), HCC (NCT03847428 and NCT03778957) and BC (NCT03395899) and in patients with untreated melanoma brain metastases (NCT03175432) or mucosal melanoma (NCT04091217).

### 2.3. Inhibitors of the Tryptophan–Kynurenine–Aryl Hydrocarbon Receptor Pathway

Tryptophan catabolism is involved in physiological immune suppression through the tryptophan–kynurenine–aryl hydrocarbon receptor (Trp–Kyn–AhR) pathway, and it plays a role in acquired and intrinsic resistance to immunotherapy [57]. Three enzymes are involved in this pathway, among which indoleamine 2,3-dioxygenase (also called as IDO1) is the most studied, while another two enzymes—tryptophan-2,3-Dioxygenase (TDO), regulated by tryptophan, cholesterols, and prostaglandin E2, and IDO2, whose activity and regulation is uncertain—play a minor role in this pathway [58].

IDO1 is ubiquitously expressed, being mostly represented in the gut, lung and genital tracts [59]. It is expressed by macrophages and DCs, and its transcription is regulated, above all, by IFNγ [59].

IDO1’s immunosuppressive activities are both direct, through the depletion of tryptophan, and indirect, with the production of metabolites like kynurenine [60]. Indeed, the depletion of this essential amino acid leads to an accumulation of empty tryptophan-tRNA, with a stress response that causes T cell anergy, while kynureninecan inhibits the activation of T cells, inducing IDO1 expression in DC and the activation of T_reg_ lymphocytes [60,61].

The physiological role of IDO1 is to induce acquired immune tolerance (i.e., towards fetal antigens during pregnancy), while this enzyme seems not to be involved in the constitutive maintenance of immune tolerance toward self-antigens [62,63].

IDO1 also plays an aberrant role in tumors. Its presence has been observed in several cancer cell lines (endometrial, CRC and melanoma), and it shows a strong correlation with a worse outcome, lower presence of tumor infiltrating lymphocytes (TILs) and high percentage of T_reg_ cells [64,65,66].

Moreover, IDO1 expression in the peritumoral stroma strongly correlates with a low immune response. High IDO1 in sentinel lymph nodes has demonstrated to be associated with a reduction in TILs and worse prognosis in melanoma patients [67]. It exerts an inhibitory role and reduces the response to both anti-CTLA-4 and anti-PD1/PDL1 therapy, while the inhibition of IDO1 expression potentiates the response to both immunotherapy and chemotherapy [68,69]. Actually, IDO knockout mice with melanoma have longer survival than wild-type ones when treated with immunotherapy, while the combination of an IDO inhibitor with cytotoxic drugs like paclitaxel or with ICIs reduces tumor growth in mice with melanoma and BC [68,69].

Based on these data, several IDO inhibitors have been developed. Among them, epacadostat (INCB024360)—an orally available, reversible competitive IDO1 inhibitor—showed potent anti-IDO1 activity in vitro, resulting in T/NK cell proliferation and T_reg_ lymphocyte suppression [70,71,72].

Results from a phase I trial, which haS enrolled 52 patients with advanced tumors, suggested that epacadostat has manageable side effects and exerts an adequate IDO1 inhibitor activity, with indirect pharmacodynamical data showing a significant reduction in plasma kynurenine levels [73]. The reported dose limiting toxicities (DLTs) were fatigue and pneumonitis; no objective responses were recorded, with stable disease (SD) as the best response in 13.5% of patients [73].

Subsequently, early phase studies combining epacadostat with other treatments were more promising. Data from the ECHO-202 and ECHO-204 Phase I/II studies showed a safe toxicity profile for the combination of epacadostat with pembrolizumab and nivolumab, respectively [74,75]. The most frequent adverse events (AEs) were skin rash and lipase elevation, with promising data regarding activity [74,75]. It is noteworthy, indeed, that ORRs of 55% for the combination of pembrolizumab and epacadostat and of 75% for the combination of nivolumab and epacadostat were achieved in the melanoma cohort with interesting, although lower, ORRs in other tumor types like head and neck (H&N) cancer, urothelial cancer and NSCLC [71,75,76].

Due to these encouraging results, a Phase III trial (ECHO-301) was conducted. It recruited 706 ICI-naïve patients with unresectable Stage III or IV melanoma [77]. They were randomized to receive epacadostat at 100 mg orally twice daily plus pembrolizumab at 200 mg every 3 weeks or a placebo plus pembrolizumab for up to 24 months [77]. This study’s results were negative; indeed, with a median follow up of 12.4 months (IQR, 10.3–14.5), there were no differences in progression free survival (PFS) (HR, 1.00; 95% CI, 0.83–1.21; one-sided *p* = 0.52) or overall survival (OS) (median not reached in either group; HR, 1.13, CI, 0.86–1.49; one-sided *p* = 0.81) [77]. The most recorded Grade 3 or worse toxicity was a lipase increase, in less than 5% of patients in both arms [77].

Several doubts regarding the ECHO-301 trial remain. The most relevant ones are if the dose used was sufficient to inhibit IDO1 activity in the TME (with pharmacodynamical data on IDO1 inhibition from Phase I trials only giving an indirect suggestion of activity) and, above all, if targeting only IDO1 is sufficient to control tryptophan catabolism or if the use of a dual inhibitor may be more successful in these patients [58].

The results of the ECHO-301 trial, however, were consistent with those reported in other Phase II and III trials conducted in different settings. In NSCLC patients (KEYNOTE-715-06/ECHO-306-06), the combination of epacadostat, pembrolizumab and platinum-based chemotherapy does not guarantee a benefit in ORR. In RCC (KEYNOTE-679/ECHO-302), no difference in ORRs were observed between pembrolizumab plus epacadostat compared to the standard of care—sunitinib or pazopanib. In recurrent or metastatic H&N squamous cell carcinoma (KEYNOTE-669/ECHO-304), the combination of epacadostat and pembrolizumab was not more powerful but was less toxic than the EXTREME regimen [78].

Beyond epacadostat, new IDO inhibitors are under clinical development, such as navoximod (NLG-919), an oral dual IDO1 and TDO inhibitor, and BMS-986205, an irreversible IDO1 inhibitor [70].

Navoximod showed an ORR of only 11% in combination with atezolizumab in heavily pretreated patients, with a safe profile [79]. On the other hand, BMS-986205, in combination with nivolumab, achieved an ORR of 34% in the advanced bladder cancer cohort of a Phase I/II trial (NCT02658890), and a Phase III trial is ongoing in muscle-invasive bladder cancer patients [80].

Taken together, these results might suggest reconsidering the IDO inhibition strategy overall.

Indeed, off-target effects of IDO inhibition—such as the prolonged activation ofAhR, the iper-activation of the mTOR pathway and the alteration of the gut microbiota—might be responsible for the lack of efficacy of these compounds [58]. On the other hand, these off-target effects could be exploited to find new powerful combinations and to implement the research on predictive biomarkers to select the most suitable population [81].

See Table 3 for ongoing clinical trials targeting this pathway.

### 2.4. The PI3K/AKT/mTOR Pathway

The PI3K/AKT/mTOR pathway is physiologically involved in multiple functions, among which cell growth and survival, metabolism and motility are the most important [82]. Briefly, this pathway is activated by growth factors via receptor-tyrosine kinases (RTK); these kinases phosphorylate phosphatidylinositol-4,5-bisphosphate (PIP2) into phosphatidylinositol-3,4,5-trisphosphate (PIP3), inducing a cascade that eventually causes the recruitment and activation of phosphoinositide 3-kinase (PI3K), phosphoinositide-dependent kinase-1 (PDK1), mammalian target of rapamycin complex 2 (mTORC2) and AKT [82]. Then, AKT activates mTORC1, which induces cell proliferation and survival and increases cell metabolism [82]. PI3Ks are divided in three classes, among which Class I is the best characterized. It is further divided into Class IA PI3Ks (PI3Kα, PI3Kβ and PI3Kδ) and Class IB PI3Ks (PI3Kγ) [83].

Beyond its role in normal cells, this pathway is also dysregulated in about 30% of cancers, mediating tumor initiation, progression and drug resistance. Several mechanisms are involved, including activating mutations of RTK, PI3K, AKT or RAS or inactivating mutations of regulatory proteins like the phosphate and tensin homologue (PTEN) [84].

Recent studies have focused their attention on the role of the PI3K/AKT/mTOR pathway in the immune response. Preclinical data suggest that an alteration of the PI3K/AKT/mTOR pathway might promote an immunosuppressive response [85].

PTEN-mutant melanoma murine models showed a higher expression of immune suppressive cytokines, a decreased infiltration of CD8^+^ T cells and a reduced T cell-mediated cytotoxicity, while treatment with a PI3Kβ inhibitor enhanced the response to both PD-1 and CTLA4 inhibitors [86]. PIK3CA-mutated bladder cancer patient-derived xenografts (PDXs) have a non-T-cell-inflamed phenotype, while the administration of BKM120, a pan PI3K inhibitor, induces immune activation and the response to PD-1 inhibitors [87].

Other evidence highlights that PDL-1 expression in tumor cells is also related to this pathway. Indeed, both PTEN-mutant triple negative breast cancer (TNBC) and CRC have higher expression of PDL-1. Moreover the use of a PI3K inhibitor or of rapamicin induces a reduction in PDL-1 expression in tumor cells [88,89,90].

The PI3K pathway also regulates MDSC differentiation. High PI3Kδ expression is related to a higher number of FOXP3^+^T_reg_ cells, while the use of an inhibitor of this pathway reduces T_reg_ cells without affecting CD8^+^ TILs and reduces tumor growth [91,92,93].

Lastly, preclinical data suggest that the PI3K/AKT pathway is involved in the metabolic shift (from glycolysis to oxidative phosphorylation) that converts effector CD8^+^ T cells to memory T cells [94] and regulates the tumor associated macrophages’ (TAMs’) switch between immune stimulation and immune suppression [95].

This pathway has already been targeted therapeutically on the basis of its pleiotropic role in several malignancies. mTOR inhibitors, PI3K inhibitors (PI3Ki), dual PI3K–mTOR inhibitors and AKT inhibitors are approved in different settings such as advanced RCC (temsirolimus and everolumus), advanced ER+ BC (alpelisib and everolimus) and chronic lymphatic leukemia (CLL) (idelalisib) [84].

Nevertheless, its role in the immune response has not been exploited until recently.

New early phase studies with combinations of ICIs and PI3k/AKT/mTOR inhibitors aim at enhancing the immune response based on the aforementioned strong preclinical rationale. Preliminary data from a Phase I trial show that ipatasertib, an AKT inhibitor, added to atezolizumab and paclitaxel or nab-paclitaxel as a first-line treatment in TNBC guarantees an ORR of 73% (95% CI, 53–88) regardless of PD-L1 status or PIK3CA/AKT1/PTEN alteration status. Moreover, the toxicity profile was acceptable, with Grade ≥3 AEs recorded in 54% of patients, the most frequent all-grade AEs being diarrhea (88%; with a grade ≥3, 19%) and rash (69%; with a grade ≥3, 27%) [96]. A Phase III study is ongoing in these patients (NCT04177108).

Other trials are actually combining drugs that target this pathway with immune checkpoint inhibitors alone (NCT03131908) or in combinations with other compounds (NCT03772561). Among them, a Phase I/II trial is exploring a PI3K inhibitor with nivolumab and ipilimumab (NCT04317105) in PI3K/AKT-mutated solid tumors, while a combination of Nab-rapamycin and nivolumab is under development in advanced sarcoma (NCT03190174) (Table 4).

### 2.5. Targeting Tumor-Associated Macrophages

Tumor-associated macrophages (TAMs) are fundamental components of the microenvironment of solid tumors. TAMs express immune checkpoint modulators including PD-L1 and chemokines such as CCL17, CCL22, CXCL10 and IL-8, which attract T_reg_s to tumor sites, downregulating the immune-response [97,98]. Although the main population of TAMs is immunosuppressive M2 macrophages, TAMs can be re-programmed into M1-type helper macrophages to enhance the antitumor effects of checkpoint inhibitors [99].

The first and most widely used therapeutic macrophage-based strategy has been TAM depletion from tumor sites via the inhibition of colony stimulating factor-1/colony stimulating factor-1 receptor (CSF-1/CSF-1R) and CCL2/CCR2 inhibition [100,101]. Despite interesting pre-clinical results in mouse models, this strategy has failed to show activity in clinical trials to date [102].

Other explored strategies are the inhibition of PI3Kγ, CCL5-CCR5 and CXCR2 but also the use of antibodies binding the macrophage-derived protein MARCO [103]. Moreover, drugs that antagonize the CD47-SIRPα pathway are under development; CD47 is a transmembrane protein both on normal and cancer cells. Its signaling is based on an anti-phagocytic response, inducing resistance to phagocytosis by macrophages. Magrolimab is a first-in-class CD47 inhibitor that has been tested in a clinical trial, but several other agents are under study now [104,105,106]. Selected ongoing trials with drugs targeting macrophage checkpoints are listed in Table 5.

## 3. Immune-Metabolism Dysregulation at Host Level

The metabolism of the host itself is physiologically involved in the immune response and can play a role in both de novo and acquired resistance to ICIs [10]. Some characteristics, like body weight and gender, seem to impact the immune response, but the data are still few and controversial [11,107]. On the other hand, the composition of the microbiome affects the efficacy of ICIs and is also an intriguing target for potentially building up and fostering the immune response in different malignancies [108,109].

### 3.1. Obesity Paradox

Excess body weight is a major public health problem worldwide. Indeed, an elevated body mass index (BMI) has been associated with reduced cancer survival, with ten obesity-related cancers listed by World Cancer Research Fund [110,111].

However, some recently published studies demonstrated that, among patients with a diagnosis of cancer, an elevated BMI is unexpectedly associated with improved survival, in the so-called “obesity paradox” [112,113,114,115,116,117,118]. The obesity paradox has been postulated in different metastatic and non-metastatic settings such as in patients with RCC and CRC receiving surgery [112,114,117] and patients affected by lymphoma who have underwent autologous hematopoietic cell transplantation [119]. Interestingly, an analysis of a cohort of 423 metastatic melanoma patients suggested a positive association between the benefit from ICIs and BMI, but it was burdened by several confounders [107]. Moreover, in a retrospective analysis of 55 NSCLC patients treated with immunotherapy, a higher BMI was related to a longer OS, while hypercholesterolemia was related to a longer PFS [120].

A possible explanation for the obesity paradox might be the immune-suppressed phenotype frequently observed in obese patients as a result of chronic inflammation, with a decrease in natural killer T cells, CD8^+^T cells and M2 macrophages and an increase in PD-1 expression [121].

Moreover, the pharmacokinetics of ICIs are extremely different from those of classical cytotoxic agents, as they are metabolized, above all, in the liver, and their metabolism might be compromised in obese patients due to the altered metabolism of free fatty acids and the presence of pro-inflammatory cytokines [107,121,122]. A plausible altered clearance, together with the diffuse use of flat doses in ICI administration and the lack of data analyzing the pharmacokinetics in patients with high BMIs make the relationship between BMI and the ICI response even more complex [107].

Lastly, some methodological considerations should be addressed. First, the time-point at which the BMI was determined during these studies is important: before the diagnosis, peri-diagnosis or post diagnosis (usually 12 months after the treatment’s start) [110,118]. Wu et al. showed a better survival outcome for patients who were overweight post-treatment but not in patients with a high pre-diagnosis BMI [123]. Age is another aspect to be considered as a potential bias factor. In fact, both Brunner et al. and Navarro et al. observed that the obesity paradox was confirmed only in younger patients and not in patients older than 60 years [115,124]. Another limitation of the use of BMI is that it does not differentiate between lean mass and fat mass. In a study enrolling 175 patients with breast, gynecological, lung, gastrointestinal and H&N carcinomas, the obesity paradox was confirmed only using BMI and not when obesity was defined using other indices such as the fat-free mass index or fat mass index [125].

### 3.2. Gender Effect

Sex chromosomes and hormones are involved in the regulation of both local and systemic determinants of carcinogenesis, such as cancer-initiating cells, the composition of the TME, cell metabolism and the immune system’s functions [126].

One of the most important differences between male and female bodies is related to the percentages of lean and fat mass: lean, and metabolically active, mass is responsible for 80% of a man’s and 65% of a woman’s body mass, although it is not a variable considered in the dosing of standard chemotherapy or ICIs [127].

Differences in the physiological immune response can justify a distinct benefit from ICIs in male and female populations. Several meta-analyses evaluated whether there were gender-related differences in the ICI response [11,126,128,129,130,131].

Four of them suggested that anti-CTLA-4 treatment might guarantee a longer survival in men but not in women [11,128,131,132].

However, the suggested absence of benefit from anti-CTLA-4 in female patients needs to be interpreted really carefully. On the one hand, the early phase of the immune response is stronger in women, both in terms of more efficient APCs and a wider population CD4 + T cells [133].

On the other hand, these results may be specific for some tumor types, not being confirmed in a population of women with melanoma [126,131].

Moreover, these meta-analyses rely on published results and not on raw data, making it difficult to control bias in the selection criteria and confounding factors like lifestyle or attitude and compliance with treatment [134].

As demonstrated by Han et al. with a rigorous computational approach, there is an unexpected divergent pattern in sex-associated differences across different cancer types (melanoma and lung cancer) [135].

Lastly, a great deal of interest was raised in biomarkers of response. Some differences based on gender have been highlighted in the neoantigen load, PD-L1 protein expression and tumor mutational burden (TMB), as well as in other immune checkpoints (e.g., LAG3 and IDO1) and the distribution of immune cell populations (active CD4/CD8 T cells, memory CD4/CD8 T cells and T_reg_ cells) [135].

These results, taken together, suggest prospective clinical trials stratified by gender in the randomization process.

### 3.3. Microbiota

The intestinal microbiota has been estimated to contain more than 100 trillion bacteria [136]. The interactions between resident microbes and the immune system have been largely explored [137].

Zitvogel et al. hypothesized that the microbiota plays a role in antitumor immunosurveillance through the cross-reactions between the microbiota and tumor antigens, the production of bacterial metabolites that might have systemic modulatory effects and the stimulation of pattern-recognition receptors (PRRs) [138].By activating PRRs, mostly expressed by innate immune effectors, microbes can stimulate the production of cytokines and interferons, which can determine the tendency to inflammatory and immunostimulatory or, on the other hand, to immunosuppressive reactions [138].

The composition of the intestinal microbiota in mouse models seems to affect the efficacy of ICIs [109,139]. In particular, Sivan et al. showed that genetically identical mice derived from two different mouse facilities and with differences in their commensal microbes showed a distinct tumor growth and response to immunotherapy, while cohousing leveled those differences [109].

Several authors have also confirmed these hypotheses in human species, reporting that a particular gut microbiota composition could affect the response to immunotherapy in different tumors such as melanoma, NSCLC, RCC, and urothelial carcinoma [108,140,141]. Frankel et al. published the first report on human gut microbiota metagenomic and metabolomic profiling in melanoma patients treated with immunotherapy, showing that ICI responders were enriched for *Bacteroidescaccae* [142]. A wide-diversified microbiota seems to be more frequent in patients with a great benefit from the treatment, with a direct correlation between a large diversification of the microbiota and a higher number of T cell numbers in both the blood and TME [108,140,141].

An interesting field of discussion is how concomitant medications can modify the gut microbiota and, eventually, the response to ICIs.

The associations between antibiotic (ATB) usage and the efficacy of immunotherapy was investigated in different studies with contradictory results [139,143,144,145]. In the retrospective study of Derosa et al., 121 patients with RCC and 239 patients with NSCLC were evaluated [144]. Patients treated with recent ATB therapy (30 or 60 days before the administration of ICIs) were compared with patients who did not receive ATBs, with the results highlighting a shorter PFS and OS for those patients receiving an ATB in both the RCC and NSCLC groups [144].

Furthermore, the combination of ATBs and proton pump inhibitors (PPI) has also been associated with gut dysbiosis, decreased bacterial richness, and the promotion of T-cell tolerance [146].

Chalabi et al. recently published an analysis including 1500 patients from randomized, controlled trials and first reported a detrimental effect of PPI and ATBs on ICI efficacy in patients with advanced NSCLC [146].

It seems that ATB treatment may reduce the efficacy of ICIs, undermining the wide array of bacterial species, each one with a distinct metabolomic and metagenomic profile [108,140,141].

Similarly, PPI affect the gut microbiome; it is suggested that they directly increase stomach pH, whose role is, physiologically, to prevent infections from food-derived and oral bacteria [147].

Lastly, the composition of the microbiota can also affect the resistance to immune-related adverse events (IrAEs) [148,149,150]. A study demonstrated that the presence of bacteria belonging to the *Bacteroidetes* phylum in the intestinal microbiota is correlated with resistance to the development of ICI-induced colitis [148]. In a prospective study, a baseline gut microbiota enriched with *Faecalibacterium* and other *Firmicutes* is associated with both a better clinical response to ipilimumab and a higher risk of ipilimumab-induced colitis [149].

To date, the available studies have been too heterogeneous, above all because it has been very difficult to use a standardized methodology in this field; therefore, further investigation regarding the role of the gut microbiota in ICIs’ resistance (and response) mechanisms are needed.

A promising therapeutic approach using the microbiota seems to be fecal microbiome transplantation (FMT), basically, a transplant of the feces from immune-responder patients.

In preclinical models, FMT in germ-free mice led to improved tumor control and a higher response to immunotherapy [108,140,141]. Phase I and II trials in melanoma patients are ongoing (NCT03353402 and NCT03341143) exploiting FMT to revert immune resistance. An ongoing early Phase 1 trial (NCT03686202) explores another approach, alternative to fecal transplantation—microbial ecosystem therapeutics (MET), a defined mixture of pure live cultures of intestinal bacterial isolates from a stool sample of a healthy donor administered with ICIs.

Other strategies such as oral supplementation with specific bacteria or antibiotic therapy might modify the intestinal microbiota in order to enhance the effect of anticancer therapies and, hopefully, in the future, to overcome resistance to ICIs [109,140].

## 4. Conclusions and Future Perspectives

The therapeutic algorithm has been massively changed by the introduction of ICIs for several malignancies (from metastatic melanoma and advanced NSCLC to RCC, urothelial cancers and squamous cell cancer of the H&N, and from hematologic cancers, such as classic Hodgkin’s lymphoma, to rare tumors such as Merkel cell carcinoma) with, eventually, a broad-range approval for solid tumors with microsatellite instability (MSI-H) and mismatch repair deficiency.

However, in this evolving field, primary and secondary resistance are an emerging issue [10]. Understanding de novo resistance may allow the discovery of predictive biomarkers of response for increasing response rates in tumors that are theoretically “cold” (i.e., intrinsically resistant), while tackling acquired resistance could prolong clinical benefit in patients who are initially responders but then progress [10].

As described above, different metabolic pathways are involved, and most of them are interdependent. Alterations of the TME, as well as mutations of cancer-related pathways, interact with host factors such gender, BMI and, above all, variations in the microbiota, modifying the immune response and, eventually, the benefit from ICIs [151,152]. These metabolic alterations are interesting targets to overcome immune resistance, but although some of them have yet achieved regulatory approval for modifying therapeutic algorithms, for others, the results are frankly disappointing or at least controversial [49,51,77].

Several points need to be taken into account when designing new clinical trials and treating patients with ICIs.

- Are there any predictive factors that should be taken into account for tailoring immunotherapy?
  1. PD-L1 expression, microsatellite instability and the tumor mutational burden have already been demonstrated to be useful biomarkers in specific settings; however, their sensitivity is limited, and their effectiveness has been confirmed only in specific types of tumor [153].
  2. Other predictive factors such as the genomic signatures and metabolic profiles associated with ICI resistance are under preclinical and clinical evaluation, and some of them have shown encouraging results [154]. The presence of mutations of Janus kinase 2 (JAK2), beta2-microglobulin and serine/threonine kinase 11 (STK11) are some of the alterations that have recently emerged as potential predictors of low responsiveness to ICIs [155,156,157].
  3. Heterozygosity in the HLA class I genotype, a characteristic that facilitates the presentation of a broader set of tumor antigens to T cells, is another possible response biomarker that has been demonstrated to confer a higher responsivity to ICIs in cancer patients [158,159].
  4. A dynamic research area aims at finding predictors of response, specifically for the combination of antiangiogenics and ICIs. In patients with HCC treated with the combination of bevacizumab and atezolizumab, there was a correlation between the expression of a signature of pre-existing immunity, including PD-L1 (HR = 0.42) and T effector markers (HR = 0.46), and a longer PFS while Notch pathway activation correlated with a worse clinical outcome [160]. Interestingly, higher levels of VEGF receptor 2 (HR = 0.36), T_reg_s (HR = 0.35), myeloid inflammation (HR = 0.43) and triggering receptor expressed on myeloid cells 1 (TREM1)/MDSC signatures (HR = 0.43) were predictive of a longer PFS in patients treated with atezolizumab and bevacizumab than in those who had received the ICI alone, suggesting a possible role for these markers [160].
  5. Among host factors, the gut microbiome composition is another potential predictor of response. This is particularly interesting because the determination of the gut microbiome does not require invasive procedures such as tumor biopsies [108,140].
- Considering host factor relevance, how should we modify both clinical trial designs and, eventually, everyday practice?
  1. Prospective clinical trials should consider a gender-based randomization approach. This may significantly contribute to a deeper comprehension of the role of gender in the antitumor activity of ICIs and help in the identification of predictors of response/resistance and toxicities to ICIs, differently expressed in men and women.
  2. Baseline BMI, not only performance status (PS), should be considered as a stratification factor in future ICI trials.
  3. The effect of antibiotics and proton pump inhibitors on the response to ICI therapy warrants further investigation. Physicians should carefully evaluate the need for co-medications such as antibiotics or proton pump inhibitors during immunotherapy.

In conclusion, metabolic pathways play a crucial, although not fully understood, role in both intrinsic and acquired resistance to ICIs.

The reformulation of prospective clinical trials, taking into account host factors, and the identification of both new predictive factors and new compounds tackling immune metabolism might be the successful tools for comprehending and overcoming resistance in the immunotherapy era.

## Figures and Tables

**Table 1:** Selected ongoing clinical trials exploring adenosine pathway.

Molecular Target	Agents	Phase	Indications	Clinical Trial Identifier	Status
**CD73**	Oleclumab + Durvalumab	II	Renal, pancreatic, head and neck and NSCLC with DNA Methylation	NCT04262375	Active not recruiting
**CD73**	Oleclumab +/− Durvalumab	I	Bladder cancer	NCT03773666	Recruiting
**CD73**	Carbo-taxol + Durvalumab +/− Oleclumab	I/II	Triple negative breast cancer (TNBC)	NCT03616886	Recruiting
**CD73**	Oleclumab + Durvalumab vs. Durvalumab + Monalizumab	II	NSCLC	NCT03822351	Recruiting
**CD39**	TTX-030 + Pebrolizumab or Chemotherapy	I	Solid tumors	NCT03884556	Recruiting
**CD73** **Anti-A2AR**	Oleclumab + AZD4635 + Osimertinib	I/II	NSCLC	NCT03381274	Recruiting
**CD73** **Anti-A2AR**	Oleclumab + AZD4635 + Durvalumab	II	Prostate cancer	NCT04089553	Recruiting
**CD39** **CD73**	IPH5201 + Oleclumab +/− Durvalumab	I	Solid tumors	NCT04261075	Recruiting

**Table 2:** Selected published clinical trials of immune checkpoint inhibitors (ICIs) in combination with bevacizumab.

ICI Agent in Combination with Bevacizumab	Tumor Type	Phase	Clinical Trial Identifier	Reference
**Atezolizumab**	NSCLC	III	NCT02366143 (IMpower 150)	Socinski et al., *Lancet Resp*, 2019 [49]
**Pembrolizumab**	NSCLC	II	NCT02039674 (Keynote-021)	Gandhi et al., *Lancet Oncol*, 2016 [52] Gandhi et al., *J Thor Oncol*, 2019 [5]
**Atezolizumab**	Hepato-cellular carcinoma (HCC)	III	NCT03434379 (IMbrave 150)	Cheng et al., *ESMO*, 2019 [50] Finn et al., *NEJM*, 2020 [51]
**Atezolizumab**	Renal cell carcinoma (RCC)	III	NCT02420821 (IMmotion 151)	Motzer et al., *Lancet*, 2019 [53]
**Atezolizumab**	RCC	II	NCT01984242 (IMmotion150)	Powles et al., *Nat Med*, 2018 [54]
**Atezolizumab**	Microsattelite-instable (MSI) mCRC	Ib	NCT01633970	Hochster et al., *JCO*, 2017 [55]
**Ipilimumab**	Melanoma	I	NCT00790010	Hodi et al., *CancerImm*, 2014 [56]

**Table 3:** Selected ongoing clinical trials exploring the tryptophan–kynurenine–aryl (Trp–Kyn–AhR) pathway.

Molecular Target	Agents	Phase	Indications	Clinical Trial Identifier	Status
**IDO-1**	Epacadostat + pembrolizumab	II	Muscle invasive bladder urothelial cancer	NCT03832673	Not yet recruiting
**IDO-1**	Epacadostat + urvalumab	II	Epstein–Barr virus-positive nasopharyngeal cancer	NCT04231864	Not yet recruiting
**IDO-1**	Epacadostat + embrolizumab	II	Esophageal and gastric tumor	NCT03196232	Recruiting
**IDO-1+ vaccines (mesotelin &GM-CSF)**	Epacadostat + embrolizumab, + RS-207 +/− yclophospamide + VAX	II	Pancreatic cancer	NCT03006302	Recruiting
**IDO-1**	Electroporetion +/− Epacadostat + embrolizumab	II	Head and neck (H&N) cancers	NCT03823131	Recruiting
**IDO-1**	Nivolumab + MS986205	II	H&N cancers	NCT03854032	Recruiting
**IDO-1**	Nivolumab + MS986205	II	Endometrial carcinoma and carcinosarcomas	NCT04106414	Recruiting
**IDO-1**	Neoadjuvantgemcitabine + cisplatin +/− Nivolumab +/− BMS 986205. Post-surgery Nivolumab +/− or BMS-986205	III	Muscle-invasive bladder cancer	NCT03661320	Recruiting

**Table 4:** Selected ongoing clinical trials exploring the PI3K/AKT pathway.

Molecular Target	Agents	Phase	Indications	Clinical Trial Identifier	Status
**AKT + PARP+** **PDL-1**	AZD5363 + laparib + urvalumab	I	Advanced solid tumors	NCT03772561	Recruiting
**AKT+** **PDL-1**	Paclitaxel +/− patasertib +/− tezolizumab	III	TNBC	NCT04177108	Recruiting
**PI3K+ PD-1+CLTA4**	Copanlisib + ivolumab +/− pilimumab	I/II	PI3K/AKT-mutated solid tumors	NCT04317105	Not yet recruiting
**PIK3b+ PD-1**	GSK2636771 + embrolizumab	I/II	PTEN-deficient melanoma	NCT03131908	Recruiting
**PIK3+PD-1**	Duvelisib + embrolizumab	I/II	Head and neck cancer	NCT04193293	Recruiting
**mTOR+PD1**	ABI-009 (Nab-rapamycin) + ivolumab	I/II	Advanced sarcoma	NCT03190174	Recruiting

**Table 5:** Selected ongoing clinical trials exploring macrophage checkpoint blockade.

**Molecular Target**	**Agents**	**Phase**	**Indications**	**Clinical Trial Identifier**	**Status**
**CSF-1R**	SNDX-6532 + Durvalumab	II	Cholangiocarcinoma	NCT04301778	Recruiting
**CSF-1R**	Cabiralizumab + Nivolumab	II	Peripheral T cell lymphoma	NCT03927105	Recruiting
**CSF-1R**	DCC-3014 + Avelumab	Ib	High grade sarcomas	NCT04242238	Recruiting
**CXCR2**	AZD5069 + Enzalutamide	II	Metastatic castration resistant prostate cancer	NCT03177187	Recruiting
**MDSCs**	SX-682 + Pembrolizumab	I	Melanoma	NCT03161431	Recruiting
**CCR5**	Leronlimab (PRO 140) + carboplatin	Ib/II	TNBC	NCT03838367	Recruiting
**CD47**	SGN-CD47M	I	Solid tumors	NCT03957096	Recruiting
**CD47**	Magrolimab + chemotherapy	I	B-cell Non-Hodgkin’s lymphoma	NCT02953509	Recruiting
